# A cross-sectional study on self-medication with over-the-counter drugs among adolescents: an analysis of current practices

**DOI:** 10.3389/fpubh.2025.1560299

**Published:** 2025-06-26

**Authors:** Chenyang Ma, Hanqing Zhang

**Affiliations:** Dalian University of Technology Central Hospital, Dalian, China

**Keywords:** self-medication, adolescents, over-the-counter drugs, drug safety, health education

## Abstract

**Background:**

Self-medication refers to individuals using over-the-counter (OTC) medications to manage self-diagnosed health issues or symptoms without professional medical guidance. Although OTC medications are generally considered safe and effective, significant knowledge gaps and safety risks persist among adolescents when using these drugs. This study aims to explore the self-medication behaviors of adolescents in China regarding OTC medications and the influencing factors.

**Objective:**

This study explores adolescent self-medication strategies for OTC drug use through multi-setting surveys in hospitals, pharmacies, and schools, aiming to enhance understanding of adolescents’ medication behaviors and improve awareness of safe medication practices.

**Methods:**

This cross-sectional study distributed data via questionnaires from adolescents aged 12 to 18 between August 2023 and August 2024. The questionnaire included demographic information, health status, OTC medication usage patterns, motivations for use, knowledge of OTC drugs, and purchasing behaviors. A total of 600 questionnaires were distributed, and after logical consistency checks, 573 valid questionnaires were included in the final analysis.

**Results:**

The study found that 97.5% of adolescents used OTC medications in the past year, with 52.5% using them 1–2 times, 31% using them 3–5 times, and 14% using them more than 5 times. However, 23.75% lacked knowledge of proper usage, 25.67% misunderstood antibiotic use, 6.67% were unaware of adverse reactions, and 9.17% did not recognize Traditional Chinese Medicine side effects.

**Conclusion:**

This study highlights common self-medication among adolescents with significant knowledge gaps on OTC drugs and side effects. It calls for better health education, especially in schools, and a more active role for pharmacists and healthcare professionals. Regulatory bodies should enhance oversight, and future research should explore OTC traditional Chinese medicines and social adaptability.

## Highlights

- The results of this study indicate that adolescents’ understanding of over-the-counter (OTC) medications needs improvement, and there are significant deficiencies in their self-medication practices. Therefore, it is essential for pharmaceutical professionals to provide health education to adolescents.
- The research data reveals that adolescents surveyed in hospitals, community pharmacies, and vocational schools have a poor understanding of the safety and contraindications of over-the-counter (OTC) medications. This finding provides valuable guidance for future health interventions.
- Policies, educational conditions, and financial support may hinder pharmacists or pharmaceutical professionals from providing health education to school-aged adolescents. Overcoming these challenges will require coordinated efforts across multiple sectors.

## Introduction

Self-medication refers to the practice of managing self-diagnosed health issues or symptoms by using medications that are approved and accessible without a prescription (over-the-counter [OTC] drugs). These medications are generally considered safe and effective when used as recommended (1, 2). Including using leftover prescriptions or altering treatment regimens (3), often based on non-professional advice. This practice can also include using leftover prescription drugs for oneself, friends, or family members, as well as altering the prescribed treatment regimen—such as extending or shortening the treatment duration, or adjusting the dosage of prescribed medications. These behaviors are all considered forms of self-medication (4). The ease of acquiring and using over-the-counter (OTC) medications without professional supervision can introduce several safety risks. For example, individuals may incorrectly self-diagnose and select inappropriate medications, potentially worsening or delaying their condition. Moreover, non-compliance with dosages can result in drug dependence, misuse, or adverse effects. These issues underscore the necessity for comprehensive education and guidance on proper medication use, despite the widespread availability of OTC drugs (5).

Drug safety involves optimizing the therapeutic benefits while minimizing associated risks. This depends on the proper indication for use, the absence of contraindications, correct dosage, and a minimal likelihood of adverse effects. Traditionally, drug safety monitoring has primarily focused on prescription medications. However, it is equally important to extend this monitoring to over-the-counter (OTC) products, which are purchased not only from pharmacies but also from non-healthcare retail outlets such as supermarkets, service stations, and online platforms (6).

The safety concerns related to self-medication with over-the-counter (OTC) drugs among adolescents are significant. Adolescents, typically defined as individuals aged 12 to 18 years (7), are influenced by several factors when engaging in self-medication behavior (6, 7). A positive attitude toward self-care, coupled with overconfidence in their knowledge of medications, often drives the practice of self-medication and contributes to drug misuse (8, 9). Common behaviors include using medications without a prescription, relying on outdated prescriptions, sharing medications with friends or family, and consuming leftover medications from previous prescriptions or household stock (10). Both OTC and prescription-only medicines (POMs) are easily accessible without adequate information on their indications and contraindications, which exposes adolescents to unnecessary risks (11, 12). While adolescents may exhibit a sense of responsibility, the risks associated with self-medication—such as misuse, overuse, or drug abuse—remain a concern (13).

Global studies highlight rising self-medication trends, with prevalence rates varying from 17 to 60% across regions (11–13). However, research in Asian contexts, especially China, remains limited. Despite notable disparities in OTC regulation and cultural attitudes toward self-care, few studies have explored Chinese adolescents’ perceptions of OTC safety or the role of social adaptability—a key factor in health decision-making (14, 15). This gap is critical, as social adaptability may influence how adolescents navigate health information and make medication choices. To address this, this study investigates the prevalence, patterns, and influencing factors of self-medication among Chinese adolescents, with a focus on integrating social adaptability as a novel dimension. By examining data from hospitals, pharmacies, and schools, we aim to identify knowledge gaps and inform interventions to promote safe OTC use (16).

The issue of self-medication among adolescents has emerged as a growing area of scientific research (17). In Western countries, there is a well-established body of work exploring the relationship between physical health and self-medication. However, studies on this subject, particularly in Asian and developing countries, remain limited. Much of the available literature on adolescents’ concerns regarding the safety and efficacy of self-medication comes from international sources. Despite notable disparities in OTC regulation and cultural attitudes toward self-care, and comprehensive studies addressing adolescents’ perceptions of the safety and efficacy of self-medication in China are currently lacking. As such, conducting research with a global perspective is crucial for understanding the interactions between adolescents’ physical health and self-medication, especially within different cultural contexts. This study seeks to address this gap by investigating the prevalence of self-medication among Chinese adolescents, examining their behavioral patterns, identifying influencing factors, and with a focus on integrating social adaptability as a novel dimension (18).

## Aim

This study aims to assess the prevalence, behavioral patterns, and influencing factors employed by adolescents with over-the-counter (OTC) drugs through surveys conducted in hospitals, community pharmacies, and schools. The results will contribute to a better understanding of adolescents’ medication practices and serve as a foundation for improving their awareness of safe medication use, as well as promoting evidence-based approaches to OTC drug use in self-medication and explore its relationship with social adaptability.

## Methods

### Study design

This study utilizes a cross-sectional design to collect data via questionnaires, aiming to examine the prevalence and influencing factors of over-the-counter (OTC) drug use among adolescents aged 12 to 18 years between August 2023 and August 2024. While this design is well-suited for describing current self-medication patterns and associations, it cannot establish causal relationships between variables, a limitation that should be considered when interpreting the results. To ensure the validity of the questionnaire, the study adapted the standardized tool developed by Smith and incorporated expert reviews during its design. The original Smith questionnaire included three core domains: general medication literacy, self-medication behaviors, and health-seeking attitudes. In adapting it for this study, we added a 4-item sub-scale on social adaptability and revised questions to reflect Chinese OTC regulatory contexts. Content validity was assessed through a two-step process: first, a panel of 5 experts (pediatricians, pharmacists, and adolescent health researchers) evaluated item relevance, leading to the removal of 2 redundant questions. Second, a pilot test with 50 adolescents confirmed the questionnaire’s reliability (Cronbach’s *α* = 0.85), meeting established psychometric standards. The questionnaire gathers information on participants’ demographics, health status, OTC drug usage patterns, motivations, perceptions of OTC drugs, and purchasing habits. Additionally, all participants provided informed consent prior to completing the survey, and the study adhered to the ethical approval requirements set by the ethics committee.

### Participants

The study gathered 600 questionnaires from adolescents aged 12 to 18 across three distinct settings: hospitals, community pharmacies, and vocational schools. These settings were selected for their representative nature. The questionnaire includes a social adaptability sub-scale adapted from the Social Adaptation Self-Evaluation Scale (SASS), which measures adolescents’ ability to seek social support, process health information, and make collaborative decisions. This sub-scale consists of 5 Likert-type items (e.g., “I can easily ask pharmacists or doctors for help with OTC drugs”), scored on a 5-point scale (1 = “strongly disagree,” 5 = “strongly agree”). Validation steps: Item-level analysis showed acceptable discrimination (item-total correlations > 0.4), and confirmatory factor analysis supported a single-factor structure (CFI = 0.92, RMSEA = 0.06), establishing construct validity. Participation was voluntary and anonymous, with no incentives provided (19).

Participants were recruited using convenience sampling, approaching adolescents aged 12–18 who visited hospitals (outpatient departments), community pharmacies, or attended vocational schools during the data collection period. In hospitals, participants were invited while waiting for consultations; in pharmacies, they were approached during OTC drug purchases; and in vocational schools, classes were selected with the assistance of school administrators. These three settings were purposefully selected to capture diverse contexts of adolescent OTC exposure: **Hospitals**: Adolescents here often have existing health concerns, making them more likely to use OTC drugs for symptom relief. This setting allows observation of self-medication behaviors alongside professional medical interactions. **Community Pharmacies**: As the primary retail channel for OTC drugs, pharmacies provide direct access to pharmacist consultations. Studying adolescents here helps assess how professional guidance (or lack thereof) influences purchasing and usage decisions. **Vocational Schools**: This setting represents a non-hospital, non-pharmacy environment where adolescents may self-medicate independently, often relying on peer or family advice. It captures daily self-care behaviors in a structured educational setting.

**Figure 1:**
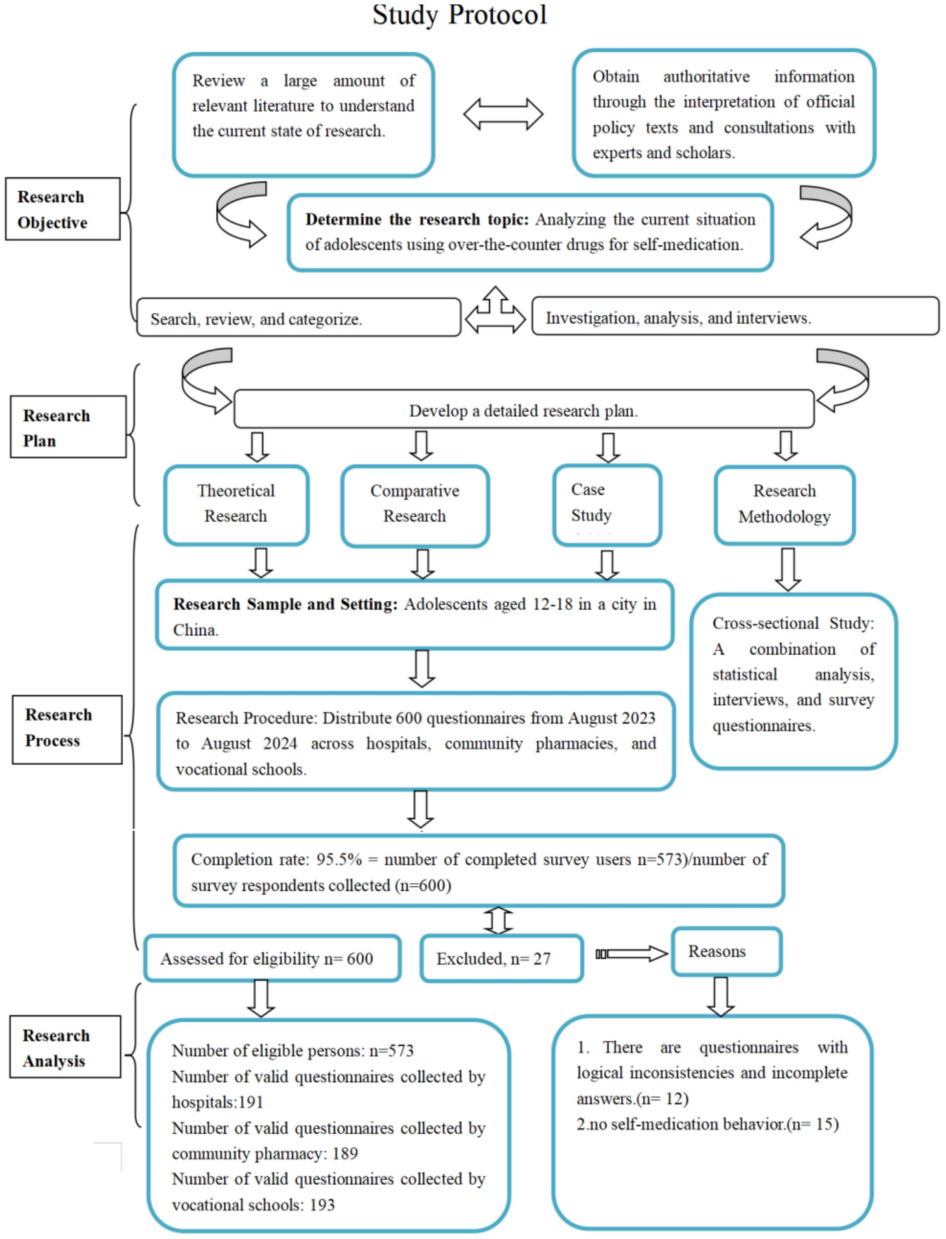
Study protocol including completion rate and participant flow chart, modified based on cross, strobe and cherries (37–39).

Together, these venues cover the continuum of OTC access—from medically oriented (hospitals) to self-reliant (schools)—ensuring a comprehensive understanding of contextual influences on self-medication. A detailed study protocol and participant flow chart are presented in Figure 1.

### Inclusion criteria

The inclusion criteria for participants in this study were as follows: (1) Participants aged 12 to 18 years. Given the possibility that respondents may report ages that differ from their actual age, 18-year-old individuals were included; (2) Participants who self-reported purchasing and using over-the-counter drugs, indicated by a “yes” response to the question on whether they had bought and used OTC medications independently; (3) Voluntary participation, with all participants completing the informed consent form; (4) Participants were able to independently complete the survey questionnaire, or seek assistance from investigators if needed.

### Exclusion criteria

(1) Individuals who are unconscious or have mental disorders.
(2) Participants in other similar research projects were also considered in this study.
(3) The questionnaire responses are incomplete or logically inconsistent.
(4) Those who have not engaged in over-the-counter self-medication.

### Data collection

From August 2023 to August 2024, a survey was conducted to collect a total of 600 questionnaires, with 200 questionnaires distributed each at hospitals, community pharmacies, and vocational schools. The focus of these questionnaires was to assess the self-medication behaviors related to over-the-counter (OTC) drugs among adolescents aged 12 to 18 years. Data was collected using anonymous self-administered questionnaires, which covered the following areas:

(1) **Demographic Information:** Age, gender, grade level, household registration, etc.
(2) **Health Status:** Recent health conditions, common ailments, etc.
(3) **OTC Drug Usage:** Frequency of OTC drug use in the past year, types of drugs used, purposes of use, etc.
(4) **Motivation for Use:** Reasons for self-medication, sources of information, etc.
(5) **Perception of OTC Drugs:** Understanding of the drugs, personal experiences with their use, awareness of associated risks, etc.
(6) **Purchasing Situation:** Methods of purchasing OTC drugs, whether professional guidance was sought, etc.

A total of 600 questionnaires were collected and subjected to logical consistency checks. Of these, 12 questionnaires with logical inconsistencies and incomplete responses were excluded, along with 15 questionnaires that did not indicate any self-medication behavior. As a result, 573 respondents were included in the study, yielding an effective response rate of 95.50%. Participation was entirely voluntary, and all participants were provided with comprehensive information about the study. By completing and returning the questionnaire, participants gave their informed consent. Confidentiality was ensured, and participants were informed that they could withdraw their consent at any time (20).

Data were collected using anonymous, paper-based self-administered questionnaires to ensure accessibility across all study settings. For participants aged 12–14 years, investigators were available to read questions aloud or clarify terminology upon request without influencing responses to minimize literacy-related bias. All data collectors (*n* = 10) received standardized training before the study, focusing on neutral questionnaire administration, handling inquiries about over-the-counter (OTC) drugs, and ensuring consistent data recording procedures across settings. Additional training was provided for collectors in hospitals and pharmacies to navigate clinical and retail environments respectfully and with minimal disruption to participants’ routines. Questionnaire completion conditions varied by setting. In schools, questionnaires were administered during 45-min class intervals (non-exam periods) under teacher supervision to ensure a quiet, distraction-free environment. Students were instructed to complete the questionnaires independently, with teachers available only to address logistical issues (e.g., page turning) but not content-related questions. In hospitals and pharmacies, participants completed the questionnaires in waiting areas or consultation rooms with investigators present to provide clarification when needed, but without direct supervision to maintain anonymity and reduce response bias. Completed questionnaires were returned sealed in opaque envelopes to ensure confidentiality, with an average completion time of 10–15 min across all settings. The original English questionnaire was translated into Chinese by two bilingual healthcare researchers and back-translated by a third expert to ensure semantic equivalence.

### Data analysis

The data analysis employed a combination of descriptive statistics, inferential statistics, and regression analysis. Descriptive statistics were used to summarize the basic characteristics of the sample and the current state of OTC drug usage. Inferential statistics were applied to examine the influence of various factors on OTC drug use. The analysis was conducted using SPSS 23.0 software, with a significance level set at 0.05. Regression analysis was performed to assess the model’s effectiveness and significance, using key metrics such as regression coefficients, standard errors, R^2^ values, and *p*-values.

## Results

### Basic characteristics of the sample

A total of 573 valid questionnaires were collected for this study. The participants’ ages ranged from 12 to 18 years, with an average age of 15.2 ± 2.7 years. Among the respondents, 47.69% were male and 52.31% were female. Students with urban household registrations comprised 55.38% of the sample, while those with rural household registrations accounted for 44.62%. All participants provided informed consent to participate in the study. The following presents the translated data from Tables 1–3, which outline the survey results on self-medication awareness among students from hospitals, community pharmacies, and vocational schools.

**Table 1:** Survey results of self-medication awareness among hospital students [*n* = 191, *n* (%)].

Serial number	Title	Yes	No	Uncertain	Accuracy %
1	Minor symptoms should be the preferred self-medication	160 (83.77)	21 (10.10)	10 (5.23)	83.77
2	Drugs are classified as prescription and over-the-counter	160 (83.77)	14 (7.33)	17 (8.90)	83.77
3	Antibiotics are over-the-counter drugs	147 (76.96)	18 (9.43)	26 (13.61)	76.96
4	Chinese patent medicines also have side effects	161 (84.29)	14 (7.33)	16 (8.38)	84.29
5	Traditional medicines are not necessarily worse than new ones	163 (85.34)	13 (6.81)	15 (7.85)	85.34
6	There are safety risks in using multiple drugs together	152 (79.58)	18 (9.42)	21 (10.10)	79.58
7	There are adverse reactions to over-the-counter drugs	163 (85.34)	12 (6.28)	16 (8.38)	85.34
8	The more expensive the medication, the safer it is	33 (17.28)	152 (79.58)	6 (3.14)	79.58
9	oral medications are safer than Injectable medications	165 (86.39)	14 (7.33)	12 (6.28)	86.39
10	The longer the course of medication, the better it is	157 (82.20)	10 (5.23)	24 (12.57)	82.20
11	Long-term use of antibiotics can cause bacterial resistance	33 (17.28)	152 (79.58)	6 (3.14)	79.58
12	When taking medicine, one should pay attention to diet	170 (89.00)	5 (2.62)	16 (8.38)	89.00
13	All the imported drugs are better than the domestic ones	76 (39.79)	112 (58.64)	3 (1.57)	58.64
14	If self-medication is ineffective, one should see a doctor	155 (81.15)	12 (6.28)	24 (12.57)	81.15
15	Self-medication can be based on personal experience to determine the dosage	19 (9.95)	160 (83.77)	12 (6.28)	83.77
16	Self-medication process can be changed at any time	34 (17.80)	148 (77.49)	9 (4.71)	77.49
17	Use multiple drugs with the same function at the same time	24 (12.57)	163 (85.34)	4 (2.09)	85.34
18	Stop taking the medicine immediately after the symptoms disappear	45 (23.56)	144 (75.39)	2 (1.05)	75.39
19	Forgetting to take the medicine can be made up by increasing the dose	41 (21.47)	147 (76.96)	3 (1.57)	76.96
20	Over-the-counter drugs can be purchased online	151 (79.06)	22 (11.52)	18 (9.42)	79.06

The average accuracy of the hospital survey results is 80.68.

**Table 2:** Survey results of self-medication awareness among community pharmacy students [*n* = 189, *n* (%)].

Serial number	Title	Yes	No	Uncertain	Accuracy %
1	Minor symptoms should be the preferred self-medication	155 (82.01)	23 (12.17)	11 (5.82)	82.01
2	Drugs are classified as prescription and over-the-counter	163 (86.24)	11 (5.82)	15 (7.94)	86.24
3	Antibiotics are over-the-counter drugs	142 (75.14)	19 (10.05)	28 (14.81)	75.14
4	Chinese patent medicines also have side effects	152 (80.43)	18 (9.52)	19 (10.05)	80.43
5	Traditional medicines are not necessarily worse than new ones	159 (84.12)	13 (6.87)	17 (8.99)	84.12
6	There are safety risks in using multiple drugs together	150 (79.37)	18 (9.52)	21 (11.11)	79.37
7	There are adverse reactions to over-the-counter drugs	151 (79.89)	22 (11.64)	16 (8.47)	79.89
8	The more expensive the medication, the safer it is	33 (17.46)	147 (77.78)	9 (4.76)	77.78
9	oral medications are safer than Injectable medications	161 (85.19)	13 (6.87)	15 (7.94)	85.19
10	The longer the course of medication, the better it is	150 (79.37)	18 (9.52)	21 (11.11)	79.37
11	Long-term use of antibiotics can cause bacterial resistance	32 (16.92)	140 (74.07)	17 (8.99)	74.07
12	When taking medicine, one should pay attention to diet	163 (86.24)	11 (5.82)	15 (7.94)	86.24
13	All the imported drugs are better than the domestic ones	74 (39.15)	112 (59.26)	3 (1.59)	59.26
14	If self-medication is ineffective, one should see a doctor	154 (81.48)	12 (6.35)	23 (12.17)	81.48
15	Self-medication can be based on personal experience to determine the dosage	25 (13.23)	149 (78.83)	15 (7.94)	78.83
16	Self-medication process can be changed at any time	23 (12.17)	155 (82.01)	11 (5.82)	82.01
17	Use multiple drugs with the same function at the same time	20 (10.58)	154 (81.48)	15 (7.94)	81.48
18	Stop taking the medicine immediately after the symptoms disappear	48 (25.40)	139 (73.54)	2 (1.06)	73.54
19	Forgetting to take the medicine can be made up by increasing the dose	45 (23.81)	140 (74.07)	5 (2.64)	74.07
20	Over-the-counter drugs can be purchased online	151 (79.90)	21 (11.11)	17 (8.99)	79.90

The average accuracy of the community pharmacy survey results is 89.02.

**Table 3:** Survey results of self-medication awareness among vocational school students [*n* = 193, *n* (%)].

Serial number	Title	Yes	No	Uncertain	Accuracy %
1	Minor symptoms should be the preferred self-medication	159 (82.38)	18 (9.32)	16 (8.30)	82.38
2	Drugs are classified as prescription and over-the-counter	160 (82.90)	15 (7.77)	18 (9.33)	82.90
3	Antibiotics are over-the-counter drugs	155 (80.31)	22 (11.40)	16 (8.29)	80.31
4	Chinese patent medicines also have side effects	152 (78.76)	21 (10.88)	20 (10.36)	78.76
5	Traditional medicines are not necessarily worse than new ones	150 (77.72)	15 (7.77)	28 (14.51)	77.72
6	There are safety risks in using multiple drugs together	157 (81.35)	12 (6.22)	24 (12.43)	81.35
7	There are adverse reactions to over-the-counter drugs	160 (82.90)	24 (12.43)	9 (4.67)	82.90
8	The more expensive the medication, the safer it is	38 (19.69)	144 (74.61)	11 (5.70)	74.61
9	oral medications are safer than Injectable medications	152 (78.76)	27 (13.99)	14 (7.25)	78.76
10	The longer the course of medication, the better it is	170 (88.08)	13 (6.76)	20 (10.36)	88.08
11	Long-term use of antibiotics can cause bacterial resistance	35 (18.13)	152 (78.76)	6 (3.11)	78.76
12	When taking medicine, one should pay attention to diet	154 (79.79)	13 (6.74)	26 (13.47)	79.79
13	All the imported drugs are better than the domestic ones	82 (42.49)	106 (54.92)	5 (2.59)	54.92
14	If self-medication is ineffective, one should see a doctor	152 (78.76)	12 (6.22)	29 (15.02)	78.76
15	Self-medication can be based on personal experience to determine the dosage	29 (15.03)	163 (84.45)	1 (0.52)	84.45
16	Self-medication process can be changed at any time	32 (16.58)	147 (76.17)	14 (7.25)	76.17
17	Use multiple drugs with the same function at the same time	31 (16.06)	156 (80.83)	6 (3.11)	80.83
18	Stop taking the medicine immediately after the symptoms disappear	62 (32.13)	128 (66.32)	3 (1.55)	66.32
19	Forgetting to take the medicine can be made up by increasing the dose	46 (23.83)	141 (73.06)	6 (3.11)	73.06
20	Over-the-counter drugs can be purchased online	156 (80.83)	11 (5.70)	26 (13.47)	80.83

The average accuracy of the vocational school survey results is 78.08.

It should be noted that in China and most other regulatory systems, antibiotics are prescription medications. However, as indicated in Table 1, a large proportion of adolescents (76.96% in hospital students, 75.14% in community pharmacy students, and 80.31% in vocational school students) misunderstood the classification of antibiotics, believing them to be over-the-counter drugs. This significant misunderstanding reflects a lack of accurate knowledge among adolescents regarding antibiotic use (Table 4).

**Table 4:** Score differences among different groups (age, gender, region, and setting).

Group classification	Sub-groups	Knowledge score (Mean ± Standard deviation)	Statistical test (*p*-value)
Age	12–14 years old	79.0 ± 11.5	0.109
	14–16 years old	81.0 ± 12.0	-
	16–18 years old	80.0 ± 12.2	-
Gender	Male	80.5 ± 11.8	0.382
	Female	81.2 ± 12.1	-
Region (classified by urban and rural areas)	Urban	81.0 ± 12.0	0.491
	Rural	80.2 ± 11.6	-
Location	Hospital	80.68 ± 12.54	0.258
	Community Pharmacy	89.02 ± 10.21	-
	Vocational School	78.08 ± 13.16	-

Knowledge scores across different groups (age, gender, region, and location), showing no statistically significant differences among these groups (*p* > 0.05 for all comparisons).

### Data analysis

**Location**: The analysis of knowledge across three locations—hospitals, community pharmacies, and vocational schools—yielded a *p*-value of 0.258, which exceeds the 0.05 threshold, suggesting that the differences in accuracy across these locations are not statistically significant.

**Gender**: The comparison of knowledge between male and female respondents resulted in a *p*-value of 0.382, which is greater than 0.05, indicating that gender does not significantly affect the accuracy of the questionnaires.

**Household Registration**: The accuracy of questionnaires for students with urban and rural household registrations returned a *p*-value of 0.491, which is above the 0.05 threshold, implying that household registration type does not have a statistically significant impact on the accuracy of the questionnaires.

**Age Group**: The analysis of knowledge across three age groups—12-14 years, 14–16 years, and 16–18 years—produced a p-value of 0.109, which is greater than 0.05, indicating that age group differences do not significantly influence the accuracy of the questionnaires.

### Current status of over-the-counter drug use

In the past year, 97.5% of adolescents reported using over-the-counter (OTC) drugs. Among these individuals, 52.5% used OTC drugs 1 to 2 times, 31% used them 3 to 5 times, and 14% used them more than 5 times. The most commonly used types of OTC medications included cold remedies, pain relievers, digestive aids, and allergy medications. In addition to these, vitamins and dietary supplements were also frequently used among adolescents, with approximately 20% of respondents reporting regular use. By age, younger adolescents (12–14 years old) showed a relatively higher frequency of using cough suppressants, likely due to more common respiratory infections at this age. In contrast, older adolescents (16–18 years old) were more likely to use energy-boosting supplements, perhaps influenced by academic pressure and increased physical activity. Regarding the relationship with time, data showed that the use of OTC medications increased slightly during the final exam periods in schools, indicating a possible association with academic stress. Seasonally, the consumption of cold remedies and anti-allergy medications peaked during the winter and spring seasons respectively, corresponding to the prevalence of colds and allergic reactions during these times. Moreover, adolescents who reported higher levels of stress, whether from schoolwork or personal life, were more likely to use OTC medications for symptom relief, although further research is needed to establish a causal relationship.

### Perception of OTC drugs

The survey on adolescents’ understanding of self-medication reveals that 79.1% of respondents have a relatively comprehensive understanding of over-the-counter (OTC) medications. However, 23.75% of adolescents are unaware of the proper usage and dosage, and 25.67% lack clarity on the correct use of antibiotics. Specifically, 25.67% of adolescents wrongly believed that antibiotics are over-the-counter drugs, which is an incorrect perception. In fact, antibiotics are prescription medications in China, and this misunderstanding can lead to serious consequences. Additionally, 6.67% of adolescents do not recognize that OTC medications may cause adverse reactions, while 9.17% are unaware that traditional Chinese medicine may also have side effects. It is a wrong belief that 6.67% of adolescents hold, as OTC medications indeed can cause adverse reactions. Similarly, the idea that traditional Chinese medicine has no side effects, which 9.17% of adolescents believe, is incorrect. The data collected from hospitals, community pharmacies, and vocational schools indicates that adolescents in these settings generally have insufficient knowledge regarding the safety and contraindications associated with OTC medications. These misunderstandings can have significant implications for self-treatment. For example, adolescents who think antibiotics are over-the-counter drugs may use them without proper medical advice when they have minor ailments. This not only fails to treat the illness effectively but also increases the risk of antibiotic resistance. Those who are unaware of OTC medications’ adverse reactions may continue to use drugs even when experiencing negative side effects, potentially worsening their health conditions. And adolescents who do not recognize the side effects of traditional Chinese medicine may misuse these medications, leading to unexpected health problems. Therefore, it is crucial to address these knowledge gaps to prevent inappropriate self-medication.

### Purchasing situation

Adolescents typically rely on various channels when purchasing over-the-counter (OTC) drugs. The main purchasing channels include:

(1) **Hospitals**: 13.24% of adolescents prefer to consult a doctor at a hospital and purchase medications, including OTC drugs, directly from the hospital pharmacy. This method is often chosen for the assurance provided by healthcare professionals.
(2) **Community Pharmacies**: A significant 62.12% of adolescents opt for community pharmacies as their primary choice for purchasing OTC drugs. These settings offer the advantage of consulting pharmacists for guidance on proper usage, dosage, and precautions, making it a popular option due to the ease of accessing professional advice without the need for a doctor’s appointment.
(3) **Online Platforms**: 22.35% of adolescents purchase OTC drugs through online platforms. Their choices are frequently influenced by advertisements or personal knowledge of the medication. While this method is convenient, it carries risks related to the potential absence of professional guidance and the influence of misleading information.
(4) **Other Sources**: A small portion, 3.56%, of adolescents rely on leftover OTC drugs from previous prescriptions or obtain them from friends. This practice carries a higher risk of misuse, as these adolescents may lack accurate information regarding the proper usage or potential side effects of the drugs.

## Discussion

### Analysis of the basic situation of adolescents’ self-medication

This study evaluated the accuracy of questionnaires collected from three locations: hospitals, community pharmacies, and vocational schools. Statistical analyses were performed across various factors, including gender, household registration type, and age groups (21). However, the results indicated that the differences in accuracy between these groups were not statistically significant, as outlined below:

**Location analysis**: The *p*-values for the questionnaires collected from hospitals, community pharmacies, and vocational schools all exceeded 0.05, indicating no significant differences in questionnaire accuracy across these locations. This suggests that the data obtained from these settings did not exhibit substantial variation. It may imply that the knowledge levels or cognitive differences among respondents in these locations were minimal, or that their demographic characteristics were relatively similar across the locations (22).

**Gender analysis**: The analysis of questionnaire accuracy between male and female respondents revealed no significant differences, with *p*-values exceeding 0.05. This indicates that gender does not significantly influence the accuracy of the questionnaires. These findings suggest that the respondents’ knowledge levels in this domain are not affected by gender-based differences (23).

**Household registration type analysis**: Similarly, no significant difference was observed in the questionnaire accuracy between students with urban and rural household registration, with *p*-values greater than 0.05. This suggests that, despite variations in living environments, the knowledge levels of urban and rural students in the areas surveyed are not significantly different (24, 25).

**Age group analysis**: When respondents were categorized into three age groups—12-14 years, 14–16 years, and 16–18 years—the p-values remained greater than 0.05, indicating no significant differences in questionnaire accuracy across the age groups. This suggests that students in these age ranges generally have a similar level of understanding of the knowledge in this area (21)^.^

### The prevalence of self-medication among adolescents and its underlying reasons

The prevalence of self-medication among adolescents and the factors driving this behavior have become a focal point in recent research. Self-medication refers to the practice of individuals selecting and using medications independently, without professional guidance, often based on personal judgment or recommendations from others. Among adolescents, self-medication is common and influenced by several key factors (26).

Firstly, many adolescents prefer managing minor illnesses or symptoms on their own, driven by a desire for independence and trust in their personal judgment. For example, when experiencing common ailments such as headaches, colds, or mild abdominal pain, adolescents often hesitate to involve their parents or seek medical attention. Instead, they opt for over-the-counter (OTC) drugs as a means to relieve their symptoms (27). Furthermore, these minor health issues are often perceived as insufficiently serious to warrant a doctor’s visit, making self-medication a more attractive option.

Secondly, the desire to save time and money plays a significant role in adolescents’ decision to self-medicate. Many adolescents view visits to the doctor or hospital as time-consuming and financially burdensome. In contrast, OTC drugs offer a more affordable and convenient alternative, providing quick symptom relief without the delays and costs associated with medical consultations (28).

Additionally, the ease of access to information is a major factor influencing self-medication among adolescents. With the proliferation of the internet, social media, and advertising, adolescents can readily access drug information. While this information is easily available, it is often not professionally vetted, which can lead to misuse or overuse of medications. Adolescents may choose medications based on advertising or personal experiences, without a full understanding of the indications, contraindications, or potential side effects (29).

Lastly, advice from family members and friends plays an important role in adolescent self-medication. Many adolescents turn to relatives or peers for recommendations on OTC drugs, especially when similar medications are already available at home (30). Although this approach is convenient, it can lead to improper medication use, particularly if the advice giver lacks accurate knowledge of the drug’s correct use.

### The potential risks of purchasing and using over-the-counter drugs

The purchasing behavior of adolescents regarding over-the-counter (OTC) drugs has drawn considerable attention in research, given its direct implications for their health and safety (31). Studies indicate that adolescents typically rely on various channels when purchasing OTC drugs (27). The most common and accessible channel is community pharmacies, where pharmacists provide guidance on the purpose, dosage, and potential side effects of medications. However, not all adolescents actively seek professional advice from pharmacists. Specifically, the survey found that only about 30% of adolescents who purchased OTC drugs at community pharmacies actively sought guidance from pharmacists. This indicates that most adolescents may lack professional medication advice when buying OTC drugs, increasing the risk of improper medication use. Some tend to rely on their own judgment or base their decisions on recommendations from family and friends. This practice can lead to the misuse or abuse of medications, particularly when there are misunderstandings about the proper indications, contraindications, or dosages (32).

Additionally, with the growing prevalence of e-commerce, an increasing number of adolescents are turning to online platforms to purchase OTC drugs. While this method offers convenience and speed, it introduces risks, such as the absence of professional pharmaceutical guidance and exposure to misleading advertisements or exaggerated claims. As a result, adolescents may overlook the potential risks associated with medications, leading to improper use (33). Further analysis revealed that adolescents who purchased over-the-counter (OTC) medications from online platforms had relatively lower knowledge scores. This indicates a close relationship between the source of purchase and adolescents’ knowledge levels as well as their rates of misuse. The lack of professional guidance associated with online purchasing channels may make adolescents more prone to medication errors.

For students in school settings, the risks associated with OTC drug use are even more pronounced due to the lack of direct access to pharmaceutical professionals. These adolescents frequently rely on advice from family members or peers, or obtain drug-related information from online sources and advertisements. However, due to their limited understanding of medications, they may remain unaware of certain side effects or contraindications, thus increasing the safety risks linked to drug use. Consequently, educating adolescents about the proper selection and use of OTC drugs is imperative (34). The misclassification of antibiotics by adolescents is a crucial issue. It must be emphasized that antibiotics are prescription drugs in China. The high prevalence of this misclassification, with 76.96% of hospital-surveyed adolescents, 75.14% of those from community pharmacies, and 80.31% of vocational school students holding incorrect views, indicates a widespread knowledge gap. Therefore, it is of utmost importance to correct adolescents’ misunderstandings about antibiotic classification and enhance their awareness of proper antibiotic use. Both schools and families must enhance their efforts to disseminate accurate drug-related knowledge to ensure the safe use of medications among adolescents.

### The necessity of strengthening health education and regulation

The results of this study show that health education provided by medical professionals, such as doctors or pharmacists, can improve the accuracy of questionnaires completed in hospitals or community pharmacies. In contrast, the accuracy of questionnaires filled out by vocational school students is relatively low (35). To mitigate the risks associated with adolescent self-medication, it is crucial to enhance health education within schools and communities, focusing on promoting proper medication knowledge. Schools should carry out systematic education programs on the safe use of OTC drugs. For example, organize a special lecture once a month, inviting professional pharmacists or doctors to explain the correct usage of OTC drugs, potential risks, and how to identify genuine and fake drugs. Conduct at least two case-analysis activities each semester to enable adolescents to deeply understand the hazards of improper medication through real-life cases. At the same time, incorporate OTC drug safety knowledge into the school health curriculum system and conduct regular assessments to ensure that students truly master the relevant knowledge. Additionally, parents and healthcare providers should take an active role in guiding adolescents toward the scientific and informed use of medications, helping to prevent reliance on blind imitation. Furthermore, government and regulatory bodies must strengthen the oversight of over-the-counter (OTC) drugs by ensuring that medication instructions are clear, accessible, and free from misleading advertising (36).

While OTC drugs can provide relief for mild symptoms, improper use poses significant risks. Adolescents, due to their limited medical knowledge, are especially vulnerable to errors in dosage, drug selection, and frequency of use. These issues can lead to overdoses, adverse drug interactions, or delays in receiving appropriate medical treatment. This concern is particularly evident with OTC traditional Chinese medicines, where instructions are often difficult to interpret. In these cases, professional guidance from a Chinese medicine specialist or a qualified pharmacist is essential to ensure proper usage. Therefore, it is imperative to strengthen the regulation and guidance of OTC drug use among adolescents to safeguard their health (32).

In addition, purchasing over-the-counter (OTC) medications online poses many regulatory challenges. At present, the regulation of online pharmaceutical markets is still not well established. Some online platforms lack strict drug admission mechanisms, leading to the proliferation of unregulated or counterfeit products. The survey shows that about (22) of adolescents are unable to distinguish whether the OTC drugs they purchase are legitimate products. This makes them highly susceptible to buying counterfeit or substandard drugs, which poses a serious threat to their health and safety. Therefore, it is urgent to strengthen the regulation of online pharmaceutical markets and improve adolescents’ ability to identify legitimate drugs.

### Enhancing the development of social adaptability

This study found that adolescent self-medication behaviors are closely linked to both physical health status and social adaptability. Consistent with previous research (Zhang et al., 2020), we found that self-medication may have negative effects on the physical and mental health of adolescents. However, this study further highlights the important role of social adaptability in this process, suggesting that adolescents with higher social adaptability are more likely to engage in rational and health-conscious behaviors. Based on this finding, we recommend incorporating social adaptability training into adolescent health education to mitigate the negative impact of self-medication behaviors.

This study has certain limitations. Firstly, the cross-sectional design was adopted. Although this design can collect a large amount of data at a specific point in time to understand the current situation of adolescents’ self-medication with OTC drugs, it cannot determine the causal relationships between various factors. For example, we cannot clarify whether self-medication behaviors lead to certain health problems or whether adolescents with health problems are more inclined to self-medicate. Secondly, the data were collected through self-reporting, which may be subject to recall bias. Adolescents may not be able to accurately recall their medication experiences, purchasing channels, and other information, which may affect the accuracy of the data. In addition, the convenience sampling method was used to select the research subjects, which may not fully represent all adolescent groups, resulting in sampling limitations and potentially affecting the generality of the research results. In future research, more rigorous research designs should be considered to address these shortcomings.

## Conclusion

This study examined the current state of over-the-counter (OTC) drug use and self-medication behaviors among adolescents in a middle school in a city in China. The findings reveal that self-medication is widespread, yet adolescents’ knowledge regarding proper drug use and potential side effects remains inadequate. The analysis of questionnaire accuracy across various locations, genders, household registration types, and age groups showed no significant differences, highlighting the need to enhance the dissemination of self-medication knowledge.

To address the health risks associated with improper medication use, it is crucial to strengthen health education initiatives targeted at adolescents, ensuring they develop a correct understanding of OTC drugs and the ability to use them safely. Additionally, greater involvement of pharmacists in providing accurate pharmaceutical information, alongside regular health education sessions in schools, is recommended. It is also essential for regulatory authorities to improve drug oversight to ensure the safe use of medications.

Future research should focus on identifying the sources of knowledge and the proper usage of OTC traditional Chinese medicines in adolescent self-medication. The innovation of this study lies in its exploration of the relationship between adolescent self-medication behaviors and social adaptation abilities, specifically within the context of Chinese adolescents. This research contributes a new perspective on adolescent health education, emphasizing the important role social adaptation plays in influencing health behaviors. Furthermore, it provides a theoretical foundation for future public health interventions, particularly in the areas of adolescent psychological support and the promotion of responsible self-medication practices.

## Data Availability

The original contributions presented in the study are included in the article/supplementary material, further inquiries can be directed to the corresponding author.

## References

[1] World Health Organization. WHO guidelines for the regulatory assessment of medicinal products for use in self-medication. Available online at: http://apps.who.int/medicinedocs/en/d/Js2218e/1.html. Accessed March 31, 2014.

[2] World Health Organization. The role of the pharmacist in self-care and self-medication. Report of the 4th WHO consultative group on the role of the pharmacist. The Hague. The Netherlands: World Health Organization (1998).

[3] Asefzadeh S Barkhordari F Moghadam F. Self-medication among cardiovascular patients of Bu-Ali hospital. J Qazvin Univ Med Sci. (2003) 7:91–4.

[4] Tajik R Shami M Mohammad Beigi A. Knowledge, attitude and practice of self-medication in mothers in Arak. Payesh Health Monit. (2011) 10:197–204.

[5] Liu D Ge P Li X Hong W Huang M Zhu L. Status of self-medication and the relevant factors regarding drug efficacy and safety as important considerations among adolescents aged 12-18 in China: a cross-sectional study. Nature Portfolio. (2024) 14:9982. 10.1038/s41598-024-59204-2 PMC11063147 38693178

[6] Bond C Hannaford P. Issues related to monitoring the safety of over-the-counter (OTC) medicines. Drug Saf. (2003) 26:1065–74. doi: 10.2165/00002018-200326150-00001, PMID: 14640771

[7] Du Y Knopf H. Self-medication among children and adolescents in Germany: results of the National Health Survey for children and adolescents (KiGGS). Br J Clin Pharmacol. (2009) 68:599–608. doi: 10.1111/j.1365-2125.2009.03477.x, PMID: 19843063 PMC2780285

[8] Stoelben S Krappweis J Kirch W. Adolescents’ drug use and drug knowledge. Eur J Pediatr. (2000) 159:608–14. 10968240 10.1007/s004310000503

[9] Hughes CM McElnay JC Fleming GF. Benefits and risks of self-medication. Drug Saf. (2001) 24:1027–37. doi: 10.2165/00002018-200124140-00002, PMID: 11735659

[10] Goldsworthy RC Mayhorn CB. Prescription medication sharing among adolescents: prevalence, risks, and outcomes. J Adolesc Health. (2009) 45:63. 19931837 10.1016/j.jadohealth.2009.06.002

[11] Ellul RD Cordina M Buhagiar A Darmanin Ellul R Fenech A Mifsud J. Knowledge and sources of information about medicines among adolescents in Malta. Pharm Pract (Granada). (2008) 6:178–86. doi: 10.4321/s1886-36552008000400002, PMID: 25157291 PMC4141727

[12] Shehnaz SI Khan N Sreedharan J. Self-medication and related health complaints among expatriate high school students: a cross-sectional survey in the United Arab Emirates. Pharm Pract (Granada). (2013) 11:211–8. 24367461 10.4321/s1886-36552013000400006 PMC3869637

[13] Loyola Filho AI Uchoa E Firmo JOA Lima-Costa MF. Estudo de base populacional sobre o consumo de medicamentos entre idosos: Projeto Bambui. Cad Saude Publica. (2005) 21:545–53. 15905917 10.1590/s0102-311x2005000200021

[14] Qato DM Alexander GC Conti RM. Use of prescription and over-the-counter medications and dietary supplements among older adults in the United States. JAMA. (2008) 300:2867–74. doi: 10.1001/jama.2008.892, PMID: 19109115 PMC2702513

[15] Oliveira MA Francisco PMSB Costa KS de Azevedo Barros MB. Automedicação em idosos residentes em Campinas, São Paulo, Brasil: prevalência e fatores associados. Cad Saude Publica. (2012) 28:335–45. 22331159 10.1590/s0102-311x2012000200012

[16] Goh LY Vitry AI Semple S Esterman A Luszcz M. Self-medication with over-the-counter drugs and complementary medications in South Australia’s elderly population. BMC Complement Altern Med. (2009) 9 10.1186/1472-6882-9-42 PMC2778637 19906314

[17] Gualano MR Bert F Passi S Stillo M Galis V Manzoli L. Use of self-medication among adolescents: a systematic review and meta-analysis. Eur J Pub Health. (2015) 25:444–50. doi: 10.1093/eurpub/cku207, PMID: 25479758

[18] da Silva C Giugliani ER. Consumption of medicines among adolescent students: a concern. J Pediatr. (2004) 80:326–32. doi: 10.2223/1208 15309236

[19] Alexa JM Bertsche T. An online cross-sectional survey of community pharmacists to assess information needs for evidence-based self-medication counselling. Int J Clin Pharm. (2023) 45:1452–63. doi: 10.1007/s11096-023-01624-7, PMID: 37532842 PMC10682211

[20] Favez L Zúñiga F Meyer-Massetti C. Exploring medication safety structures and processes in nursing homes: a cross-sectional study. Int J Clin Pharm. (2023) 45:1464–71. doi: 10.1007/s11096-023-01625-6, PMID: 37561370 PMC10682270

[21] Jung YM. Data analysis in quantitative research In: Handbook of research methods in health social sciences. Singapore: Springer (2019). 109–23.

[22] Kostyk A Zhou W Hyman MR. Using “surveytainment” to counter declining survey data quality. J Bus Res. (2019) 95:211–9. doi: 10.1016/j.jbusres.2018.10.024

[23] Liu M Wronski L. Examining completion rates in web surveys via over 25,000 real-world surveys. Soc Sci Comput Rev. (2018) 36:116–24. doi: 10.1177/0894439317695581

[24] Kato T Kishida N Umeyama T Jin Y Tsuda K. A random extraction method with high market representation for online surveys. Int J Bus Innov Res. (2020) 22:569–84. doi: 10.1504/IJBIR.2020.109036

[25] Kachroo P Kachen S. Item placement for questionnaire design for optimal reliability. J Mark Anal. (2018) 6:120–6. doi: 10.1057/s41270-018-0039-5

[26] Kaur M Al-Whaib R. Prevalence of self-medication practices and contributing factors in adolescents. J Public Health Res. (2021) 30:444–51.

[27] Marwah MK Al-Whaib R Mekkawy M Shokr H. Patterns of drug utilization and self-medication practices: a cross-sectional study. Pharmacy (Basel). (2023) 11:183. doi: 10.3390/pharmacy11060183, PMID: 38133458 PMC10747327

[28] Skarstein R. Over-the-counter medication use among adolescents in Scandinavian countries: a longitudinal study. BMC Public Health. (2019) 19:435–42.31023286

[29] Abraham A Chmielinski P. Over-the-counter analgesics: a meta-synthesis of pain self-management among adolescents. Pain Manag Nurs. (2018) 19:57–78. 10.1016/j.pmn.2021.04.010 34127393

[30] Mavletova A. Factors influencing self-medication in adolescents: the role of internet access and family influence. Soc Sci Comput Rev. (2019) 37:203–21.

[31] Innovations in Pharmacy. Opportunities for pharmacist-led education on OTC drug safety for adolescents. Innovations Pharm. (2022) 9:4.

[32] Journal of Pharmaceutical Policy and Practice. A mixed-methods systematic review of the prevalence, reasons, associated harms, and risk-reduction interventions of OTC medicines misuse, abuse, and dependence. (2023). 10.1186/s40545-021-00350-7 PMC8439034 34517925

[33] Ino H Nakazawa E. Over-the-counter drug misuse and dependence: public health ethics’ foray into fight against the codeine crisis. Pharmacy. (2022) 10:155. doi: 10.3390/pharmacy10060155, PMID: 36412831 PMC9703961

[34] American Academy of family physicians. Abuse of over-the-counter medications among teenagers and young adults. AAFP (2022). 22010610

[35] The pharma journal: Over-the-counter (OTC) drug regulation and safety. (2022).

[36] Inappropriate self-medication among adolescents. PLoS One, (2020). 10.1371/journal.pone.0189199 PMC5730183 29240799

[37] Sharma A Minh Duc NT Luu Lam Thang T Nam NH Ng SJ Abbas KS. A consensus-based checklist for reporting of survey studies (CROSS). J Gen Intern Med. (2021) 36:3179–87. doi: 10.1007/s11606-021-06737-1, PMID: 33886027 PMC8481359

[38] von Elm E Altman DG Egger M. The strengthening the reporting of observational studies in epidemiology (STROBE) statement: guidelines for reporting observational studies. J Clin Epidemiol. (2008) 61:344–9. doi: 10.1016/j.jclinepi.2a.2007.11.008 18313558

[39] Eysenbach G. Improving the quality of web surveys: the checklist for reporting results of internet e-surveys (CHERRIES). J Med Internet Res. (2004) 6. doi: 10.2196/jmir.6.3.e34, PMID: 15471760 PMC1550605

